# Comparative genomic analysis of *Aeromonas dhakensis* and *Aeromonas hydrophila* from diseased striped catfish fingerlings cultured in Vietnam

**DOI:** 10.3389/fmicb.2023.1254781

**Published:** 2023-09-22

**Authors:** Vera Irene Erickson, Le Minh Khoi, Yaovi Mahuton Gildas Hounmanou, Tu Thanh Dung, Tran Minh Phu, Anders Dalsgaard

**Affiliations:** ^1^Department of Veterinary and Animal Sciences, University of Copenhagen, Copenhagen, Denmark; ^2^Department of Aquatic Pathology, Can Tho University, Can Tho, Vietnam; ^3^Department of Aquatic Product Processing, Can Tho University, Can Tho, Vietnam

**Keywords:** *Aeromonas dhakensis*, motile *Aeromonas* septicemia (MAS), striped catfish (Pangasianodon hypophthalmus), comparative genomics, aquaculture, *Aeromonas hydrophila*

## Abstract

**Introduction:**

Motile *Aeromonas* septicemia (MAS) is a burden for striped catfish (*Pangasius hypophthalmus*) farmers in Vietnam. MAS can be caused by several species of *Aeromonas* but *Aeromonas hydrophila* is seen as the leading cause of MAS in aquaculture, but recent reports suggest that *A. dhakensis* is also causing MAS.

**Methods:**

Here we investigated the bacterial etiology of MAS and compared the genomic features of *A. hydrophila* and *A. dhakensis*. We collected 86 isolates from diseased striped catfish fingerlings over 5 years from eight provinces in Vietnam. Species identification was done using PCR, MALDI-TOF and whole genome sequence (WGS). The MICs of commonly used antimicrobials was established. Thirty presumed *A. hydrophila* isolates were sequenced for species confirmation and genomic comparison. A phylogenetic analysis was conducted using publicly available sequences and sequences from this study.

**Results:**

A total of 25/30 isolates were *A. dhakensis* sequence type (ST) 656 and 5/30 isolates were *A. hydrophila* ST 251. Our isolates and all publicly available *A. hydrophila* isolates from Vietnam belonged to ST 251 and differed with <200 single nucleotide polymorphisms (SNP). Similarly, all *A. dhakensis* isolates from Vietnam belonged to ST 656 and differed with <100 SNPs. The *tet(A)* gene was found in 1/5 *A. hydrophila* and 19/25 *A. dhakensis*. All *A. hydrophila* had an MIC ≤2 mg/L while 19/25 *A. dhakensis* had MIC ≥8 mg/L for oxytetracycline. The *floR* gene was only found in *A. dhakensis* (14/25) which showed a MIC ≥8 mg/L for florfenicol. Key virulence genes, i.e., *aerA*/*act*, *ahh1* and *hlyA* were present in all genomes, while *ast* was only present in *A. dhakensis*.

**Discussion:**

This study confirms previous findings where *A. dhakensis* was the dominating pathogen causing MAS and that the importance of *A. hydrophila* has likely been overestimated. The differences in antimicrobial susceptibility between the two species could indicate a need for targeted antimicrobial treatment plans. The lipopolysaccharide regions and outer membrane proteins did not significantly differ in their immunogenic potentials, but it remains to be determined with *in vivo* experiments whether there is a difference in the efficacy of available vaccines against *A. hydrophila* and *A. dhakensis*.

## 1. Introduction

*Aeromonas* spp. are Gram-negative bacteria belonging to the family *Aeromonadaceae*. They are commonly found in aquatic environments but can also be isolated from a variety of other sources (Janda and Abbott, 2010; Khor et al., 2018). Today, there are 36 species of *Aeromonas* and many are capable of causing disease in animals and humans. *Aeromonas* spp. are important pathogens in aquaculture, i.e., *Aeromonas hydrophila*, *Aeromonas caviae*, *Aeromonas veronii*, *Aeromonas dhakensis* and *Aeromonas salmonicida* all cause diseases in global aquaculture productions (Fernández-Bravo and Figueras, 2020). *A. salmonicida* infects cold-water fish whereas the other mentioned *Aeromonas* spp. are mesophilic and motile bacteria infecting warm-water farmed fish and less often humans (Fernández-Bravo and Figueras, 2020).

Vietnam is one of the world’s leading exporters of fish, ranking as the third biggest exporter of aquatic animal products in 2020 after China and Norway. In 2020, the value of exported aquaculture products from Vietnam reached USD 8.5 billion (FAO, 2022). Frozen striped catfish (*Pangasius hypophthalmus*) fillets are undoubtedly the most important exported product and Vietnam is the leading producer and exporter of farmed striped catfish, e.g., to China and the United States of America (FAO, 2022). Striped catfish is mainly cultured in the Mekong Delta in southern Vietnam where the production has intensified and grown fast during the last decade. The vast majority of striped catfish farmers experience one or often more disease outbreaks during the 9-month production cycle (Hoa et al., 2021). The most frequently occurring disease in striped catfish farming is motile *Aeromonas* septicemia which causes big economical losses for the aquaculture sector, not only in Vietnam but globally (Stratev and Odeyemi, 2017). Motile *Aeromonas* septicemia is caused by motile *Aeromonas* spp., mainly *A. hydrophila*, *A. caviae*, *A. veronii* and *A. dhakensis*, but variations in the prevalence of each species occur depending on the geographical region and cultured aquatic species (Stratev and Odeyemi, 2017; Azzam-Sayuti et al., 2021; Saharia et al., 2021). The symptoms of motile *Aeromonas* septicemia include anorexia, hemorrhage of internal organs and red coloring of the skin due do hemorrhage (Nielsen et al., 2001).

*A. hydrophila* has been considered the most predominant species causing motile *Aeromonas* septicemia and was detected in 11% of larvae, in 30% of fry and 30% of apparently healthy striped catfish fingerlings (Hoa et al., 2021). However, already in the beginning of the 2000’s, it was questioned whether the importance of *A. hydrophila* as a disease causing pathogen had been overestimated (Nielsen et al., 2001). With new and more precise molecular identification methods, new species of *Aeromonas* diverged and isolates previously characterized as *A. hydrophila* were re-identified as other or new *Aeromonas* spp. (Beaz-Hidalgo et al., 2013). In 2002, *A. dhakensis* was described for the first time under the name *Aeromonas hydrophila* subsp*. dhakensis* when isolates from children with diarrhea in Bangladesh showed distinctively different biochemical properties compared to *A. hydrophila* (Huys et al., 2002). Later, *A. hydrophila* subsp*. dhakensis* was elevated to the rank of species based on phylogenetic and multilocus phylogenetic analysis, and it is today considered a distinct species of *Aeromonas*, called *A. dhakensis* (Beaz-Hidalgo et al., 2013). *A. dhakensis* is described as both a human clinical pathogen and a pathogen in aquaculture. *A. dhakensis* was recently described as the most frequently isolated *Aeromonas* spp. (43%) from cultured freshwater fish farming in Malaysia, followed by *A. veronii* (22%) and *A. hydrophila* (20%) (Azzam-Sayuti et al., 2021). A study analyzing samples collected between 2013–2019 from striped catfish in the Mekong Delta area in Vietnam reports that 75% of the suspected *A. hydrophila* isolates were identified as *A. dhakensis* using different identification methods (Bartie et al., 2023).

*A. dhakensis* continues to be misidentified as *A. hydrophila* due to the application of phenotypic identification methods not able to differentiate between the two species (Chen et al., 2016). Matrix-Assisted Laser Desorption/Ionization Time Of Flight (MALDI-TOF) is a method which technically would be able to identify *A. dhakensis* at species. However, the current commercially available MALDI-TOF databases does not include *A. dhakensis*, but includes *A. hydrophila*, *A. jandaei*, *A. punctata* (*caviae*), *A. sobria* and *A. salmonicida*. Thus the current databases will give the closest matching bacterial species, i.e., *A. hydrophila* or only identify the isolate on genus level. The most accurate methods identifying *A. dhakensis* is by sequencing the housekeeping genes (*rpoB*, *rpoD* or *gyrB*) and whole genome sequence analysis (Chen et al., 2016).

Due to the relatively new taxonomy of *A. dhakensis* as a separate species and the lack of accurate identification methods in diagnostic laboratories, the real prevalence of *A. dhakensis* as a disease-causing pathogen in striped catfish is unknown. Thus the significance of *A. hydrophila* is likely overestimated, at least in Vietnamese striped catfish aquaculture (Chen et al., 2016; Lau et al., 2020; Bartie et al., 2023). The objective of this study was to conduct a comprehensive comparative analysis of the virulence *in silico*, prevalence, and genetic characteristics of *A. dhakensis* and *A. hydrophila* isolates obtained between 2017 and 2021 from striped catfish fingerlings showing symptoms of motile *Aeromonas* septicemia in eight different provinces of the Mekong Delta. By investigating key virulence factors, antimicrobial resistance profiles, and genomic traits, we aimed to elucidate the unique contributions of each species to the occurrence and severity of motile *Aeromonas* septicemia in striped catfish fingerlings. Through this comparative analysis, we have provided valuable insights into the epidemiology these emerging pathogens, ultimately contributing to the sustainable aquaculture practices and the health of striped catfish production in Vietnam.

## 2. Materials and methods

### 2.1. Specimens and bacterial isolation

**Figure 1:**
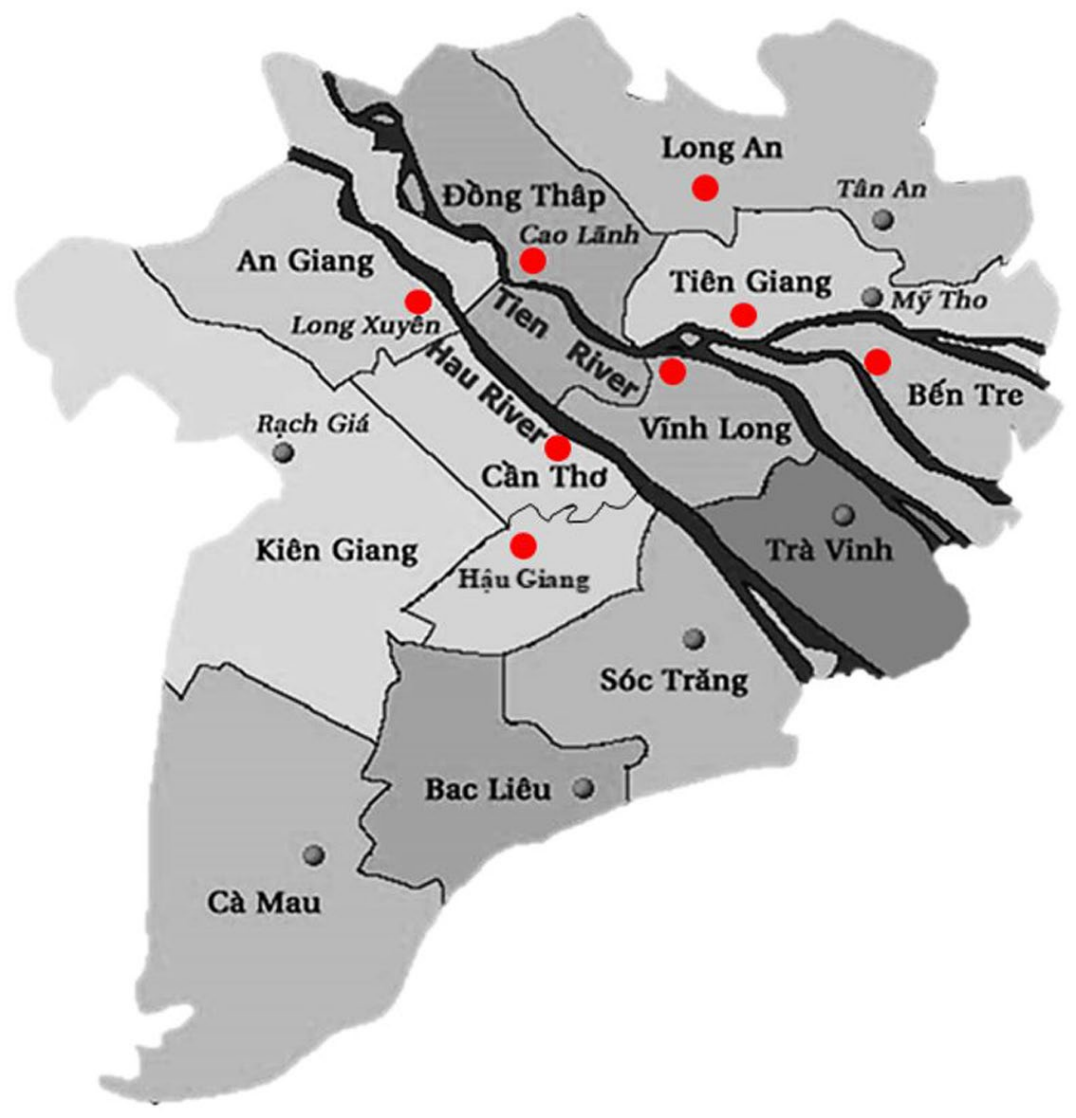
Map showing the location of provinces (red dots) where strains were collected. Province borders are marked with thin black lines. The main river branches of the Mekong Delta are marked with thick black color.

We collected diseased *P. hypophthalmus* fingerlings (size 10-100 g) and larger fish (size 100-800 g) from grow-out farms showing clinical signs of motile *Aeromonas* septicemia, i.e., pin-point hemorrhagic red spots scattered in the whole body, exophthalmia, pink-red fluid in the abdomen and enlarged spleen, liver and kidneys. Fish were anesthetized with Aqui-S^®^ (Bayer, Vietnam) and the skin was disinfected with alcohol 70% before performing necropsy. Samples from the liver and kidneys were taken using sterile inoculation loops that were cultured onto tryptic soya agar plates (TSA, Merck, Germany) and incubated at 28°C for 24 h at Can Tho University, Vietnam. Representative colony isolates on TSA plates were re-streaked onto new TSA plates to ensure purity. Isolates were initially characterized by morphology, Gram staining, catalase, oxidase, and O/F test and subsequent identified by API 20E kit (bioMérieux, France). A total of 86 bacterial isolates were collected from commercial striped catfish farms (pond sizes ranged from 0.5–1.2 ha and water depth from 4–5.5 m) located in eight different provinces in the Mekong Delta in southern Vietnam, i.e., An Giang (15 strains), Ben Tre (5 strains), Can Tho (13 strains), Dong Thap (39 strains), Hau Giang (1 strain), Long An (5 strains), Tien Giang (4 strains) and Vinh Long (4 strains) provinces. The sample locations are shown in Figure 1. Bacterial specimens were collected between 2017 and 2021 by the Department of Aquatic Pathology, College of Aquaculture and Fisheries at Can Tho University. Isolates were stored in tryptic soya broth (TSB, Merck, Germany) containing 25% glycerol and kept at –80°C before shipment to the University of Copenhagen, Denmark, for further characterization.

### 2.2. Bacterial species identification

At the University of Copenhagen material from each vial with a bacterial isolate was streaked onto agar plates with blood agar base and 5% sterile bovine blood and incubated overnight at 28°C to confirm the purity. The species of all 86 isolates was determined using *A. hydrophila* species specific PCR primers: aerolysin *aer* gene 5’CCA AGG GGT CTG TGG CGA CA 3′ (forward read) and 5′ TTT CAC CGG TAA CAG GAT TG 3′ (reverse read) with expected size of 209 bp amplicons (Pollard et al., 1990). To confirm results from the PCR, fresh bacterial colonies from all isolates were cultured on agar plates and their identity confirmed by using the automated VITEK® MS (bioMérieux, France) MALDI-TOF mass spectrometry.

### 2.3. Antimicrobial susceptibility testing

The minimum inhibitory concentration (MIC) of each isolate was determined by microbroth dilution. Each bacterial strain was streaked onto agar plates with blood agar base and 5% sterile bovine blood and incubated overnight at 37°C. Tubes with 5 mL demineralized water and tubes with 11 mL Mueller-Hinton (Oxoid) broth were prepared and incubated overnight to reduce the risk of contamination. Characteristic colonies (1 to 3) were suspended in the tubes containing demineralized water. Using a nephelometer, the bacterial concentration was measured to 0.5 McFarland Standard. Now 10 μL of the suspension was pipetted into the tube with Mueller-Hinton broth and vortexed. The Mueller-Hinton broth was poured onto an empty Petri dish and gently mixed on the table. A multi-channel pipette was used to suspend 50 μL in each well on Sensititre plates. The MIC testing was performed using the commercial Sensititre kit (AVIAN1F) from ThermoScientific™ containing the following antimicrobials: enrofloxacin, gentamicin, ceftiofur, neomycin, erythromycin, oxytetracycline, tetracycline, amoxicillin, spectinomycin, sulphadimethoxine, trimethoprim/sulfamethoxazole, florfenicol, sulphathiazole, penicillin, streptomycin, novobiocin, tylosin tartrate, and clindamycin. *Escherichia coli* ATCC25922 was used as control strain. Results were interpreted using the Thermo Scientific™ Sensititre™ SWIN™ Software System. There are no clinical breakpoints established for neither *A. hydrophila* nor *A. dhakensis* in aquaculture and therefore the epidemiological cutoff values for enrofloxacin, gentamicin, oxytetracycline and florfenicol, established by Clinical and Laboratory Standards Institute (CLSI) guidelines VET04 third edition (Clinical and Laboratory Standards Institute, 2017), for *A. hydrophila* were used for the relevant antimicrobials.

### 2.4. Whole genome sequencing

A total of 30 isolates were selected for whole genome sequencing based on results from the PCR and MALDI-TOF species identification. Only isolates confirmed as *A. hydrophila* from the MALDI-TOF analysis were chosen. Sampling year and location were considered so that isolates from all sampling years and provinces were represented. Isolates were cultured on blood agar and incubated at 37°C for 24 h. A pure bacterial colony was transferred to Luria broth (Oxoid) and incubated at 37°C for 13 h. Genomic DNA was extracted using the bacterial DNA extraction kit Maxwell^®^ RSC Cultured Cells DNA Kit (Promega Corporation, Madison, WI, United States). Quality parameters of the extracted DNA was assessed using NanoDrop™ One (Thermo Fisher Scientific, Waltham, MA, United States) and the DNA concentration using Qubit 2.0 Fluorometer (Invitrogen, Thermo Fisher Scientific, Waltham, MA, United States). Whole genome sequencing was performed with Illumina MiSeq (Illumina Inc., San Diego, CA, United States) at Statens Serum Institut (Copenhagen, Denmark) using the Nextera XT DNA Library Preparation Kit (96 samples) (Illumina Inc., San Diego, CA, United States), generating minimum sequence coverage of 50X.

### 2.5. Bioinformatic analysis

Raw sequence reads were processed for quality control using FastQC version v0.11.9. The raw reads are available at the European Nucleotide Archive (ENA) under the project number PRJEB59357. Trimming raw sequences was done with Trimmomatic version 0.3 (Bolger et al., 2014). Cleaned reads were assembled using the SPAdes 3.9 assembly tool (Nurk et al., 2013). For quality control of the assembled genomes, we used QUAST version 5.2 (Mikheenko et al., 2018). The number of contigs in each genome ranged from 125–234 contigs and the total size of the genomes was 4.85–5.09 Gbp. The assembled genomes were then subjected to further analyses. Confirmation of species identity was performed using Kraken2 (Wood and Salzberg, 2014) and KmerFinder version 3.2 (Hasman et al., 2014; Larsen et al., 2014; Clausen et al., 2018).

Multi-locus sequence typing (MLST) was done using PubMLST (Jolley et al., 2018). Screening for antimicrobial resistance genes was done in the Comprehensive Antibiotic Resistance Database Resistance Gene Identifier (CARD RGI) (Alcock et al., 2020). The selection criteria was set to perfect and strict hits. We screened the draft genomes for virulence genes in the Virulence Factor Data Base (VFDB) using the VFanalyzer tool (Liu et al., 2019) and blasted the genomes against seven additional virulence genes relevant to *Aeromonas* spp. downloaded from NCBI (*ahpA*, *alt*, *dns*, elastase, *gcaT*, *lip* and *ser*) using MyDbFinder.^1^ The threshold for ID% was set to 90% and the minimum length to 60%. To detect plasmids, we uploaded the assembled genomes to PlasmidFinder 2.1 in CGE (Camacho et al., 2009; Carattoli et al., 2014) using the Enterobactericeae database set at 95% threshold for minimum identity and minimum 60% for coverage.

The sequences were then analyzed for single nucleotide polymorphism along with publically available genomes. A search for all available *A. hydrophila* and *A. dhakensis* sequences was conducted in National Center for Biotechnology Information (NCBI), and all sequences, where at least the isolation source was available, were used in the phylogenetic analysis. Additionally, the collection date and geographical location was included in the metadata when available. Single-nucleotide variants were called by using Snippy version 4.6.0^2^ under the following parameters: mapping quality of 60, a minimum base quality of 13, a minimum read coverage of four, and a 75% concordance at a locus. Since species confirmatory results showed that among the 30 sequenced assumed *A. hydrophila* most strains (25 strains) were in fact *A. dhakensis*, two separate phylogenetic analyses were performed with *A. dhakensis* (GeneBank: CP084351.1) and *A. hydrophila* (GeneBank: CP005966.1) used as references during variant calling and alignment. The core genome single-nucleotide variants were aligned with Snippy-core version 4.1.0 for phylogeny inference.

Putative recombinogenic regions were detected and masked using Gubbins version 2.4.1 (Croucher et al., 2015). A maximum-likelihood phylogenetic tree was build using RAxML version 8.2.12 and the generalized time-reversible model with 200 bootstraps (Stamatakis, 2014). The final trees were rooted on the reference genomes and the trees were annotated and visualized with iTOL version 3 (Letunic and Bork, 2021). To support these results we constructed single nucleotide polymorphism matrices.

All of the 30 genomes were annotated using Prokka version 1.14.5 (Seemann, 2014) and the output master annotation files (.gff) were used to run a pan genome pipeline using Roary version 3.13.0 (Page et al., 2015). The pan genome was visualized in Phandango (Hadfield et al., 2018). The unique gene regions were first extracted and then analyzed in VRprofile (Li et al., 2018). Annotation was also done with Rapid Annotations using Subsystems Technology (RAST) to compare genes coding for capsular and extracellular polysaccharides (Aziz et al., 2008; Overbeek et al., 2014; Brettin et al., 2015). Since the currently used vaccine against motile *Aeromonas* septicemia is from inactivated bacteria cultures (PHARMAQ AS, Norway), we decided to further investigate any differences between the LPS and the outer membrane proteins of *A. dhakensis* and *A. hydrophila*. The analysis can provide information of potential species level differences in these regions that may have an influence on antigenic ability and vaccine efficacy. The sequences of six outer membrane proteins from *A. hydrophila* were downloaded from NCBI (GenBank: HQ326181.1, DQ177328.1, OM912661.1, HF546053.1, OM912660.1 and JQ349084.1) and combined into one multifasta file. This multifasta file was used as the reference in MyDbFinder version 2.0 (see footnote 1) with the threshold for %ID set to 90% and minimum length set to 60%. We then blasted the reference file against 5 genomes of each species. The similarities of the lipopolysaccharide (LPS) regions of each species were compared by downloading the *A. hydrophila* LPS region sequenced by Jimenez et al. (2008) and comparing 5 genomes of each species to the downloaded sequences using the Gview server^3^ The LPS sequences were downloaded from NCBI (GenBank: EU296246.1, EU296247.1 and EU296248.1).

## 3. Results

### 3.1. Species identification

**Figure 2:**
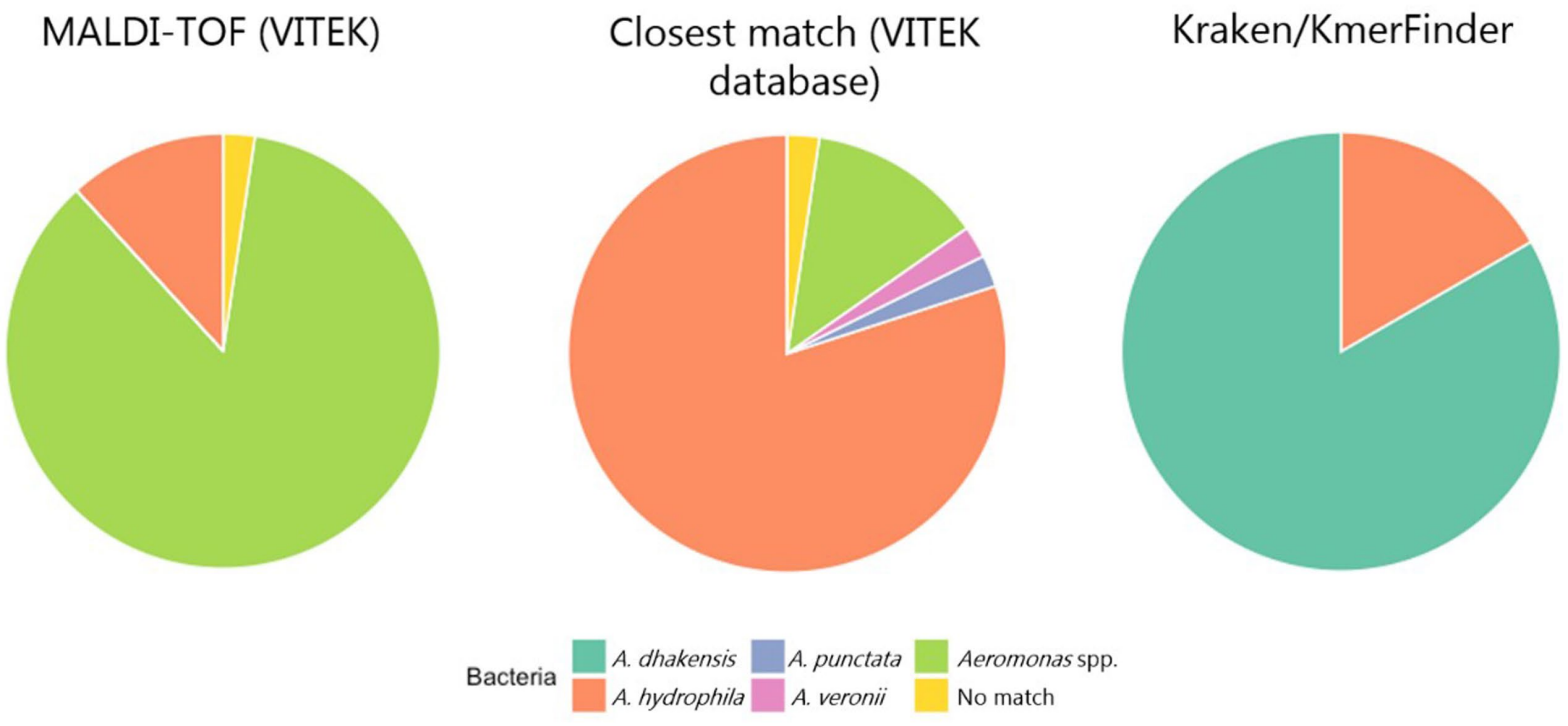
The proportion of identified *Aeromonas* spp. based on MALDI-TOF, looking for the closest matching species in the VITEK database and analysis of the sequenced genomes using KmerFinder/Kraken2.

All isolates were Gram-negative, catalase positive, oxidase positive, O/F positive and rod-shaped. The PCR analysis showed that many isolates lacked the 209 base pairs size amplicon band indicative of *A. hydrophila*. We therefore decided to run all isolates through MALDI-TOF to confirm the species. From the MALDI-TOF run, 83/85 isolates were identified as the genus *Aeromonas*. On species level, 10/85 isolates were identified as *A. hydrophila*, 2/85 had “no match” and 73/85 isolates were identified only at genus level as *Aeromonas* spp. Looking closer at the MALDI-TOF results to determine which *Aeromonas* spp. was the closest match, 2/85 isolates matched with *A. punctata*, 2/85 matched *A. veronii*, 68/85 matched closest with *A. hydrophila* and 11/85 isolates were only identified on genus level (Figure 2). Based on these results and considering sampling year and sampling location, a total of 30 isolates confirmed as *A. hydrophila* at species level were chosen for whole genome sequencing.

### 3.2. Genomic characteristics of *Aeromonas dhakensis* and *Aeromonas hydrophila*

The 30 sequenced isolates were from the following provinces: An Giang (3), Ben Tre (2), Can Tho (5), Dong Thap (9), Hau Giang (1), Hiep Thanh Com (1), Long An (4), Tien Giang (2) and Vinh Long (4). Years of isolation of the strains included: 2017 (1), 2018 (2), 2019 (15), 2020 (6) and 2021 (6). The genome GC content ranged from 60.7–61.3%. After sequencing the selected isolates, the first analysis performed was species identification based on the k-mers in the DNA sequence data using KmerFinder. KmerFinder identified 5/30 genomes as *A. hydrophila* and 25/30 isolates as *A. dhakensis*. These results were confirmed by the results from Kraken. Multi-locus sequence typing grouped all *A. dhakensis* isolates into sequence type (ST) 656 and all *A. hydrophila* genomes into ST 251 (Table 1). The genomes of the *A. hydrophila* isolates were larger than the genomes of *A. dhakensis* as they were ≥ 4.91 Gbp and the *A. dhakensis* genomes were all <4.91 Gbp. The GC content of *A. dhakensis* was ≥61.1%, while the GC content of *A. hydrophila* was ≤61.1%. Details about the 30 sequenced isolates and their genomes can be seen in Table 1. The sequences have been deposited in ENA with the project accession number PRJEB59357.

**Table 1:** Data on *A. hydrophila* and *A. dhakensis* isolates and their whole genome sequences.

Isolate	Province	Year	Weight (g)	GC (%)	Contigs	Size (Gbp)	Bacterial species	ST
AH01	Vinh Long	2019	100	61.2	167	4.86	*A. dhakensis*	656
AH02	Vinh Long	2018	30	61.3	159	4.89	*A. dhakensis*	656
AH03	Can Tho	2019	90	61.3	166	4.86	*A. dhakensis*	656
AH04	Can Tho	2019	50	61.2	135	4.87	*A. dhakensis*	656
AH05	Vinh Long	2018	500	61.3	125	4.89	*A. dhakensis*	656
AH06	An Giang	2019	400	61.2	155	4.87	*A. dhakensis*	656
AH07	Vinh Long	2019	100	61.3	166	4.87	*A. dhakensis*	656
AH09	Ben Tre	2019	50	61.2	130	4.87	*A. dhakensis*	656
AH10	Ben Tre	2019	50	61.2	146	4.87	*A. dhakensis*	656
AH13	Dong Thap	2019	80	61.3	136	4.86	*A. dhakensis*	656
AH15	An Giang	2019	70	60.7	188	5.08	*A. hydrophila*	251
AH28	Dong Thap	2017	100	61.3	134	4.85	*A. dhakensis*	656
AH41	An Giang	2019	120	61.2	142	4.87	*A. dhakensis*	656
AH42	Can Tho	2019	55	61.3	128	4.86	*A. dhakensis*	656
AH45	Dong Thap	2019	40	61.2	174	4.87	*A. dhakensis*	656
AH47	Tien Giang	2019	40	61.3	159	4.89	*A. dhakensis*	656
AH58	Can Tho	2019	30	61.3	141	4.86	*A. dhakensis*	656
AH59	Tien Giang	2019	100	61.2	142	4.87	*A. dhakensis*	656
AH61	Dong Thap	2020	60	60.7	234	5.09	*A. hydrophila*	251
AH63	Dong Thap	2020	200	61.3	139	4.86	*A. dhakensis*	656
AH70	Dong Thap	2020	NA	61.1	144	4.89	*A. dhakensis*	656
AH72	Dong Thap	2020	NA	60.7	173	5.09	*A. hydrophila*	251
AH75	Dong Thap	2020	NA	61.2	135	4.87	*A. dhakensis*	656
AH80	Dong Thap	2021	700	61.1	188	4.91	*A. hydrophila*	251
AH81	Long An	2021	70	60.7	172	5.08	*A. hydrophila*	251
AH82	Long An	2021	200	61.1	173	4.95	*A. dhakensis*	656
AH84	Can Tho	2021	100	61.2	155	4.89	*A. dhakensis*	656
AH85	Long An	2021	50	61.1	153	4.90	*A. dhakensis*	656
AH86	Long An	2021	50	61.3	168	4.86	*A. dhakensis*	656
AH87	Hau Giang	2020	15	61.3	159	4.86	*A. dhakensis*	656

Weight (g), weight of the diseased fish; ST, sequence type.

### 3.3. Phylogenetic analysis

**Figure 3:**
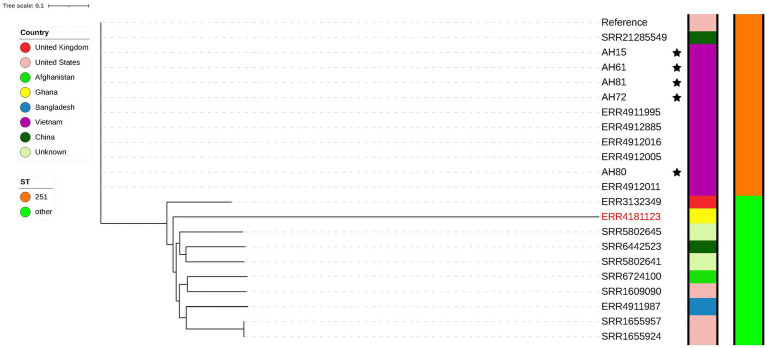
Phylogenetic tree showing strains of *A. hydrophila*. The genomes of the five strains sequenced in this study are marked with a star. A genome later confirmed as *A. dhakensis* is marked in a red colored font.

Two separate phylogenetic trees were constructed for *A. hydrophila* and *A. dhakensis*. Figure 3 shows the tree constructed using all *A. hydrophila* strains sequenced in this study and 16 publicly available *A. hydrophila* genomes downloaded from ENA. The reference strain was *A. hydrophila* (ST 251) isolated from channel catfish in the United States. The single nucleotide polymorphism (SNP) analysis revealed that one of the strains from ENA had over 200,000 SNPs comparing it to all other strains. Assembly and species analysis of the strain in KmerFinder and pubMLST revealed that this strain is in fact *A. dhakensis* ST 335. The species of all other isolates were confirmed as *A. hydrophila* with all strains originating from striped catfish in Vietnam. There were nine different sequence types and one unknown sequence type among the publicly available genomes. The SNP matrix (Supplementary Table S1) shows how these strains from Vietnam group and differ in less than 200 SNPs, including the strains sequenced in this study. All strains have ST 251, also known as virulent *A. hydrophila*. One channel catfish isolate from China (SRR21285549) closely resembles the Vietnamese strains with less than 140 SNPs and also belongs to ST 251. This strain and all the Vietnamese strains closely resemble the reference strain with less than 140 SNPs which supports the results from the sequence type analysis as all of these genomes are ST 251.

**Figure 4:**
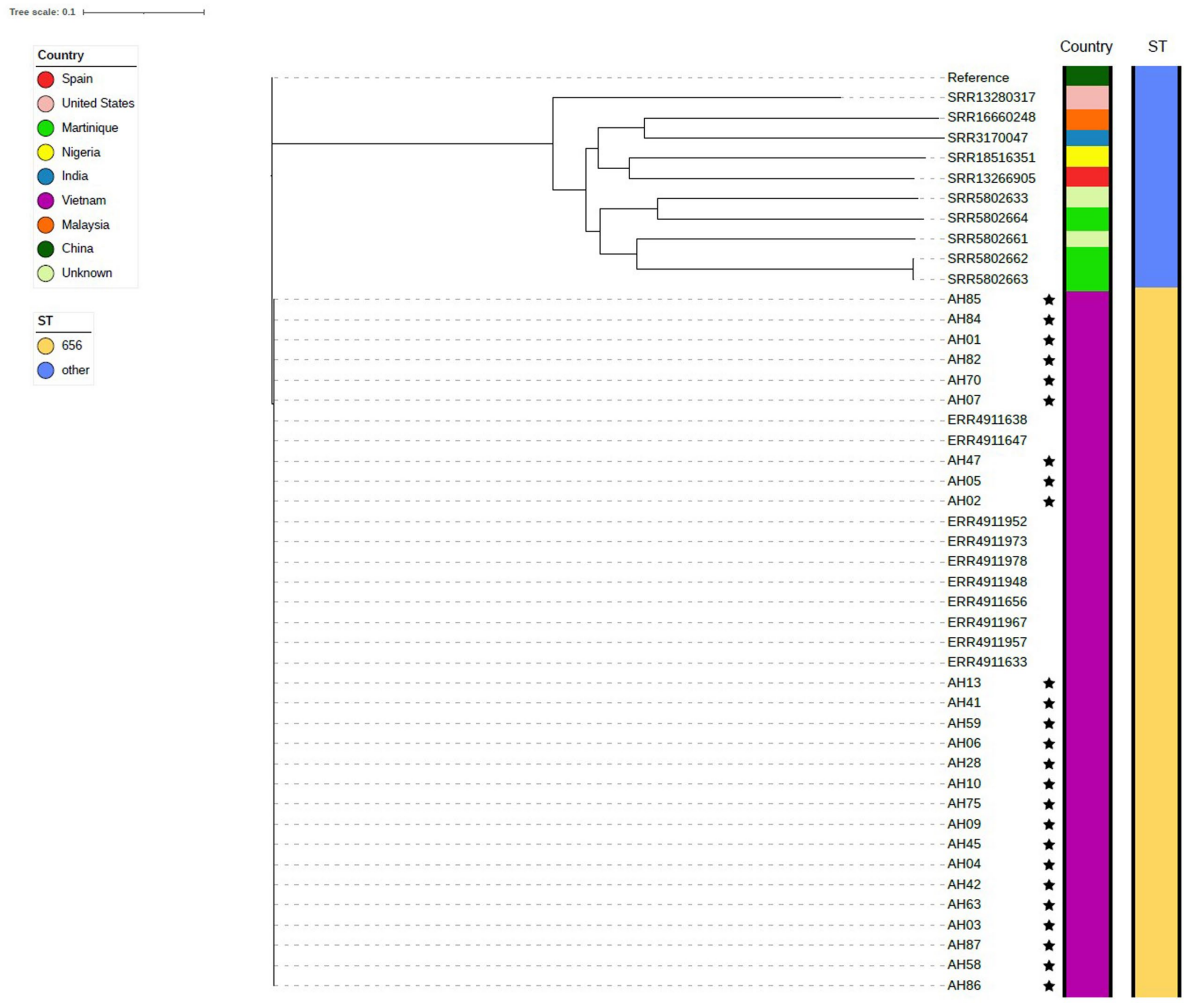
Phylogenetic tree showing isolates of *A. dhakensis*. The genomes sequenced in this study are marked with a star.

The phylogenetic tree composed of *A. dhakensis* genomes seen in Figure 4 was generated using the 25 sequenced *A. dhakensis* genomes and 20 publicly available *A. dhakensis* genomes accessed from ENA. The reference strain was isolated from a human clinical sample from China. The sequence types and species of all publicly available *A. dhakensis* genomes were confirmed using KmerFinder and pubMLST. Our isolates had a maximum difference of 108 SNPs and all belonged to ST 656. One isolate (ST 337) originating from a striped catfish in Malaysia did not group with the other isolates from the same region. The rest of the isolates from Southeast Asia (all from Vietnam) belonged to ST 656 with a difference in SNPs between any isolate being maximum 98 SNPs. Among the publicly available *A. dhakensis* genomes there was a variation in the sequence types with isolates representing eight different sequence types and two unknown sequence types. The number of SNPs between the isolates sequenced for this study and isolates from outside of Vietnam was 65,000–68,000 SNPs. The publicly available *A. dhakensis* isolates had a difference of 80,000 to 94,000 SNPs when compared to our Vietnamese isolates. The SNP matrices are available in the Supplementary Tables S1, S2.

### 3.4. Antimicrobial susceptibility, antimicrobial resistance genes and plasmids

**Figure 5:**
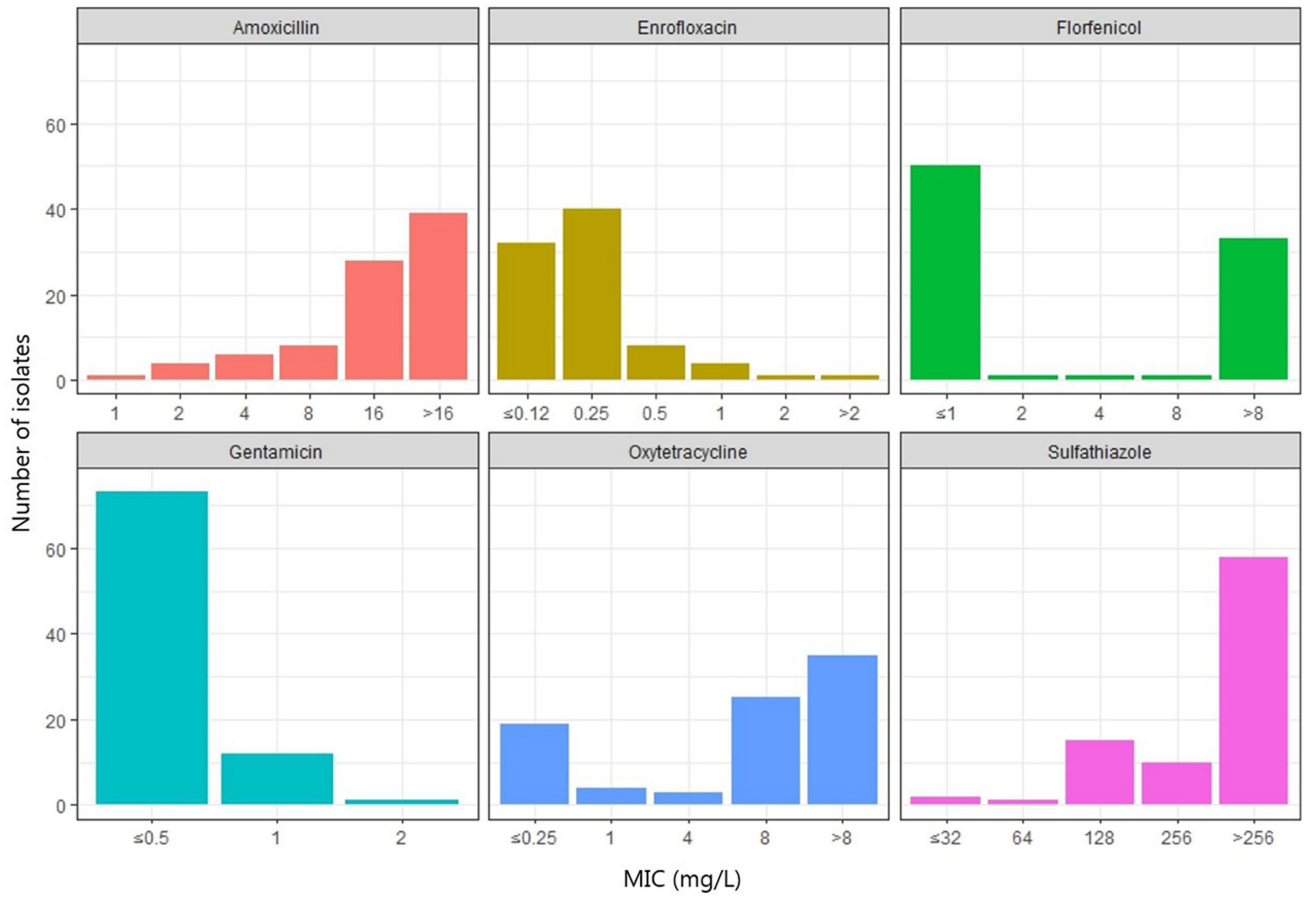
Minimum inhibitory concentrations (mg/L) of six antimicrobials commonly used for treatment of motile *Aeromonas* septicemia in striped catfish production in Vietnam.

All *A. hydrophila* isolates had an MIC ≤2 mg/L for tetracycline and oxytetracycline. Four out of five isolates were considered wild type for tetracyclines (≤ 0.25 mg/L). Most *A. dhakensis* isolates (19/25) had an MIC ≥8 mg/L for tetracycline and oxytetracycline. Six *A. dhakensis* isolates were wild type for tetracyclines. All *A. hydrophila* isolates had an MIC ≤1 mg/L for florfenicol while 11/25 *A. dhakensis* isolates had an MIC ≤1 mg/L and were wild type for florfenicol, the rest of the *A. dhakensis* isolates (14/25) had an MIC ≥8 mg/L and. The MIC for sulphathiazole was ≤128 mg/L for all *A. hydrophila* isolates and only two *A. dhakensis* isolates had an MIC <256 mg/L. All *A. hydrophila* were wild type (≤ 0.12 mg/L) for enrofloxacin, while 20/25 of the *A. dhakensis* isolates were non-wild type for enrofloxacin. All isolates, both *A. dhakensis* and *A. hydrophila*, were wild type for gentamicin (MIC ≤2 mg/L). The MIC values of the most commonly used antimicrobials in Vietnamese aquaculture production are summarized in Figure 5. The MIC values of all isolates (n = 86) for all tested antimicrobials can be found in the Supplementary Table S3.

**Figure 6:**
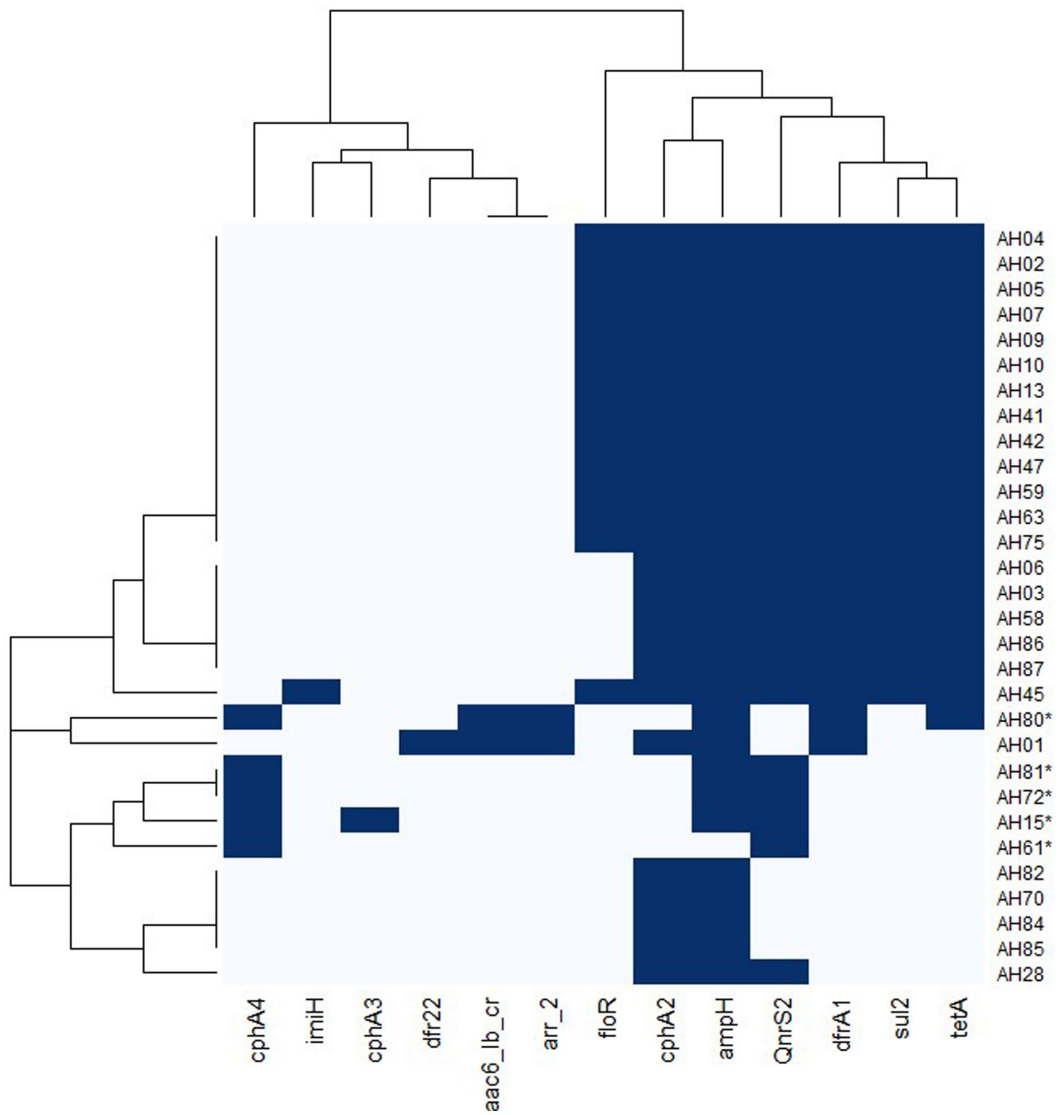
Heatmap of antimicrobial resistance genes in *A. dhakensis* and *A. hydrophila*. Dark blue color indicates presence of a gene and light blue color indicates absence of the gene. The *A. hydrophila* isolates are marked with an asterisk.

There were no plasmids detected in any of the genomes. We found the sulfonamide resistance genes *sul1* (70%) and *sul2* (63%), the quinolone resistance genes *QnrS2* (62%) and *aac(6′)-Ib-cr* (7%). Beta-lactam resistance encoding genes included *ampH* (97%) and *imiH* (3%), *cphA2* (83%), *cphA4* (17%) and *cphA3* (3%). The *cphA* genes can also code for carbapenemase resistance. The trimethoprim resistance genes *dfrA1* and *dfr22* were found in 70 and 3% of the isolates, respectively. The tetracycline resistance gene *tet(A)* was present in 67% of the genomes. Resistance genes to phenicols; *floR* (47%) and rifampin, *arr-2* (7%) were also found. All of the *A. dhakensis* genomes carried the *cphA2* resistance gene but no other *cphA* genes, while all the *A. hydrophila* genomes carried *cphA4* or *cphA3*. Apart from the genome of isolate AH80 that carried the *sul1* gene, none of the other *A. hydrophila* genomes carried genes coding for sulfonamide resistance. Twenty *A. dhakensis* isolates carried *sul1* and 19 isolates carried *sul2*. The *floR* gene was only found in *A. dhakensis*. Apart from isolate AH80, genes encoding resistance to trimethoprim, tetracycline and phenicol were not present in the *A. hydrophila* isolates. A heatmap for antimicrobial resistance genes is provided in Figure 6.

### 3.5. Virulence factors and genes in *Aeromonas dhakensis* and *Aeromonas hydrophila*

All *A. hydrophila* genomes carried virulence genes coding for Flp type IV pili. None of the *A. dhakensis* genomes carried any genes coding for this type of pili. All genomes carried genes coding for mannose sensitive hemagglutinin (Msh) pilus, polar flagella, Tap type IV pili and type I fimbriae. For type I fimbria, all genomes carried *fimC*, *fimD* and *fimF*. Only some genomes carried *fimA* and *fimE*. The type 2 and type 6 secretions systems (T2SS and T6SS) were present in all genomes, while the type 3 secretion system (T3SS) was absent in all genomes. The aerolysin *aerA*/cytotoxic enterotoxin *act*, extracellular hemolysin AHH1 gene *ahh1* and the hemolysin HlyA gene *hlyA* were present in all genomes. The gene coding for heat-stable cytotonic enterotoxin, *ast*, was only present in *A. dhakensis* genomes. The detailed results from the virulence gene analysis can be found in the Supplementary Table S4. The presence of seven genes (*ahpA*, *alt*, *dns*, elastase, *gcaT*, *lip* and *ser*) relevant for virulence in *Aeromonas* spp. was investigated in all genomes. The *ahpA* gene was present in all *A. dhakensis*, but none of the *A. hydrophila* genomes. The *alt*, *dns*, elastase and *lip* genes were present in all genomes. The *ser* gene was present in all *A. hydrophila*, but none of the *A. dhakensis* genomes.

### 3.6. Comparison genomics of *Aeromonas hydrophila* and *Aeromonas dhakensis*

#### 3.6.1. Mobile genetic elements and phages

The results from MobileElementFinder showed that insertion sequences were present in all genomes. The results from this analysis are summarized in Table 2. In *A. dhakensis*, the insertion sequences IS5, ISAhy1 and ISAs17 were present in all genomes. In *A. hydrophila*, the insertions sequences IS6100 and ISAeme7 were found in all five genomes. Only one insertion sequence, ISAeme20, was present in both species. Two types of composite transposons were found in the genomes. The transposon cn_16212_IS5 was found in two *A. dhakensis* genomes and cn_5119_ISVsa3 was found in three *A. hydrophila* genomes.

**Table 2:** Mobile genetic elements present in *A. dhakensis* and *A. hydrophila*.

Type	Name	Identity	Species	Number
Composite transposon	cn_16212_IS5	0.99	*A. dhakensis*	2
Insertion sequence	IS5	0.96–0.99	*A. dhakensis*	5
Insertion sequence	ISAhy1	0.94–0.97	*A. dhakensis*	5
Insertion sequence	ISAs17	0.97	*A. dhakensis*	5
Insertion sequence	ISAs25	1.00	*A. dhakensis*	4
Insertion sequence	ISAs29	0.96	*A. dhakensis*	1
Insertion sequence	ISAve4	0.92	*A. dhakensis*	1
Composite transposon	cn_5119_ISVsa3	1.00	*A. hydrophila*	3
Insertion sequence	IS26	1.00	*A. hydrophila*	4
Insertion sequence	IS6100	1.00	*A. hydrophila*	5
Insertion sequence	ISAeme7	0.90	*A. hydrophila*	5
Insertion sequence	ISVsa3	1.00	*A. hydrophila*	4
Insertion sequence	ISAeme20	0.99	*A. hydrophila* & *A. dhakensis*	2

The results from PHASTER showed that *A. dhakensis* only carried prophage regions of questionable completeness while *A. hydrophila* had intact prophage regions in every genome. In the *A. dhakensis* genomes there were five different prophage regions while there were two different prophage regions present in the *A. hydrophila* genomes. Table 3 summarizes the prophage region findings.

**Table 3:** Prophage regions present in selected *A. dhakensis* and *A. hydrophila* genomes.

Isolate	Completeness	Score	Total proteins	Most common phage	GC %
AH01	Questionable	85	17	PHAGE_Vibrio_fs1_NC_004306(4)	49.5%
	Questionable	80	15	PHAGE_Entero_mEp235_NC_019708(2)	49.5%
AH02	Questionable	80	29	PHAGE_Escher_D108_NC_013594(11)	58.6%
AH03	Questionable	80	14	PHAGE_Shigel_SfIV_NC_022749(2)	49.5%
AH04	Questionable	90	17	PHAGE_Shigel_SfII_NC_021857(2)	50.0%
AH05	Questionable	90	32	PHAGE_Escher_D108_NC_013594(11)	58.5%
	Questionable	80	14	PHAGE_Entero_mEp235_NC_019708(2)	49.5%
AH15^*^	Intact	100	33	PHAGE_Entero_Mu_NC_000929(10)	56.8%
	Questionable	70	13	PHAGE_Acinet_vB_AbaM_ME3_NC_041884(3)	49.2%
AH61^*^	Intact	100	33	PHAGE_Entero_Mu_NC_000929(10)	56.8%
AH72^*^	Intact	100	33	PHAGE_Entero_Mu_NC_000929(10)	56.8%
AH80^*^	Intact	100	33	PHAGE_Entero_Mu_NC_000929(10)	56.8%
AH81^*^	Intact	100	33	PHAGE_Entero_Mu_NC_000929(10)	56.8%
	Questionable	80	20	PHAGE_Acinet_vB_AbaM_ME3_NC_041884(4)	50.1%

*Aeromonas hydrophila* genomes are marked with an asterisk.

#### 3.6.2. Comparison of outer membrane proteins and lipopolysaccharide regions

We blasted the sequenced genomes against a reference sequence of six outer membrane proteins (ompA, ompC, ompTS, ompK, omp38 and omp48,) from *A. hydrophila*. *A. hydrophila* and *A. dhakensis* genomes shared outer membrane proteins. The proteins ompA and ompTS were present in all *A. hydrophila* genomes and all *A. dhakensis* genomes. The ompC (25/25) and omp48 (12/25) proteins were only present in *A. dhakensis* genomes. The omp38 protein was present in all *A. hydrophila* genomes (5/5) but was not present in any of the *A. dhakensis* genomes. The protein ompK was not present in either species. The complete results from the outer membrane protein analysis can be found in Supplementary Table S5. We also looked at differences between the genomes in RAST. When comparing the genes coding for capsular and extracellular polysaccharides the only difference between *A. dhakensis* compared to *A. hydrophila* were the genes coding for rhamnose containing glycans and dTDP-rhamnose synthesis.

All five *A. hydrophila* genomes from this study and five *A. dhakensis* genomes were compared to the LPS region displayed by *A. hydrophila* AH-3 (Jimenez et al., 2008). In both species, the genes *kdkA*, *waaC*, waaA, wahF, *waaE*, *waaF* and *coaD* were similar to the LPS region in isolate AH-3. The region containing the *hldD*, *wahA*, *waaL*, *wahB*, *wahC* and *wahE* varied between the two species (Supplementary Figure S1).

#### 3.6.3. Pan genome analysis and analysis of species-specific coding sequences

**Figure 7:**
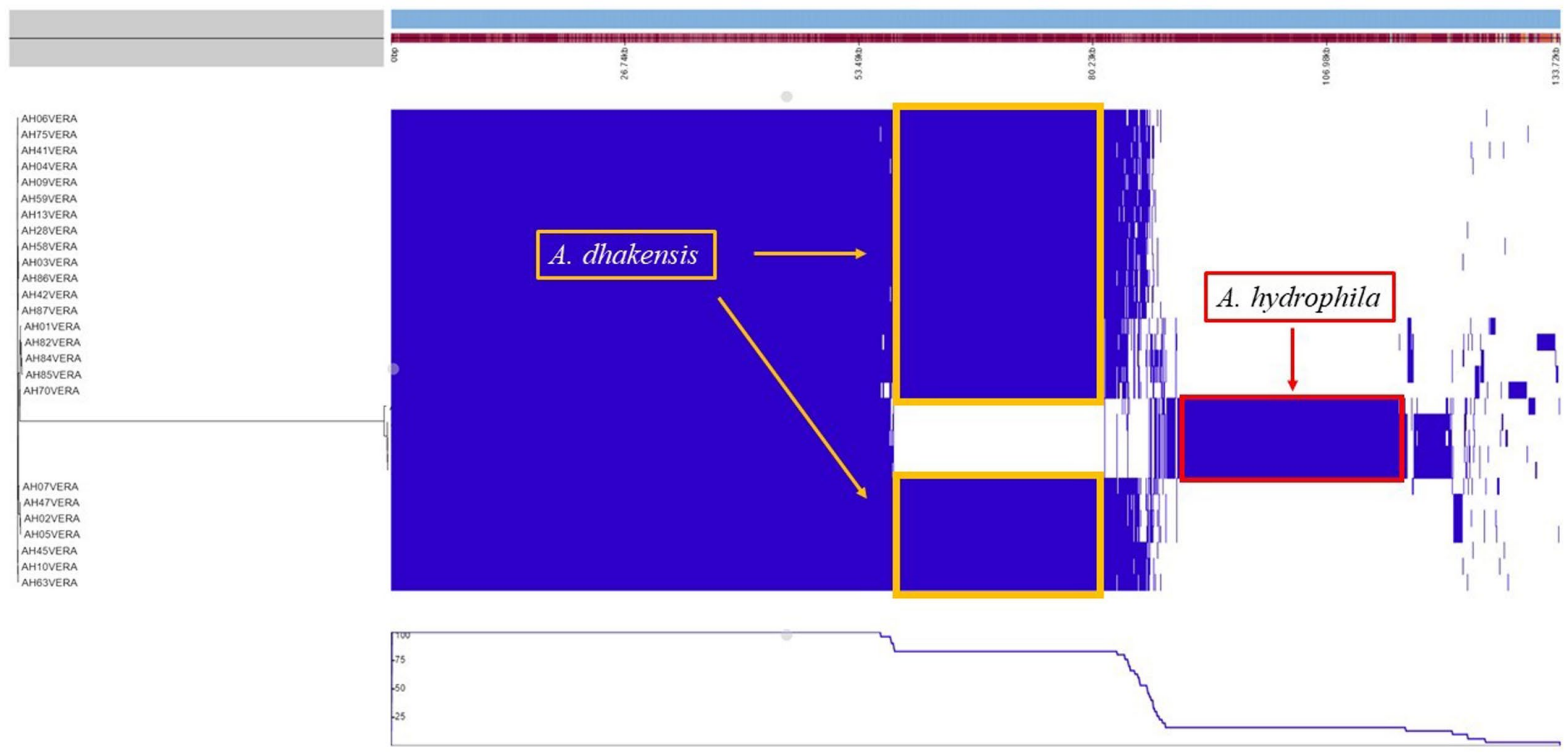
Pan genome analysis of *A. hydrophila* and *A. dhakensis.* The unique regions for each species are marked with yellow (*A. dhakensis*) and red (*A. hydrophila*) color.

**Figure 8:**
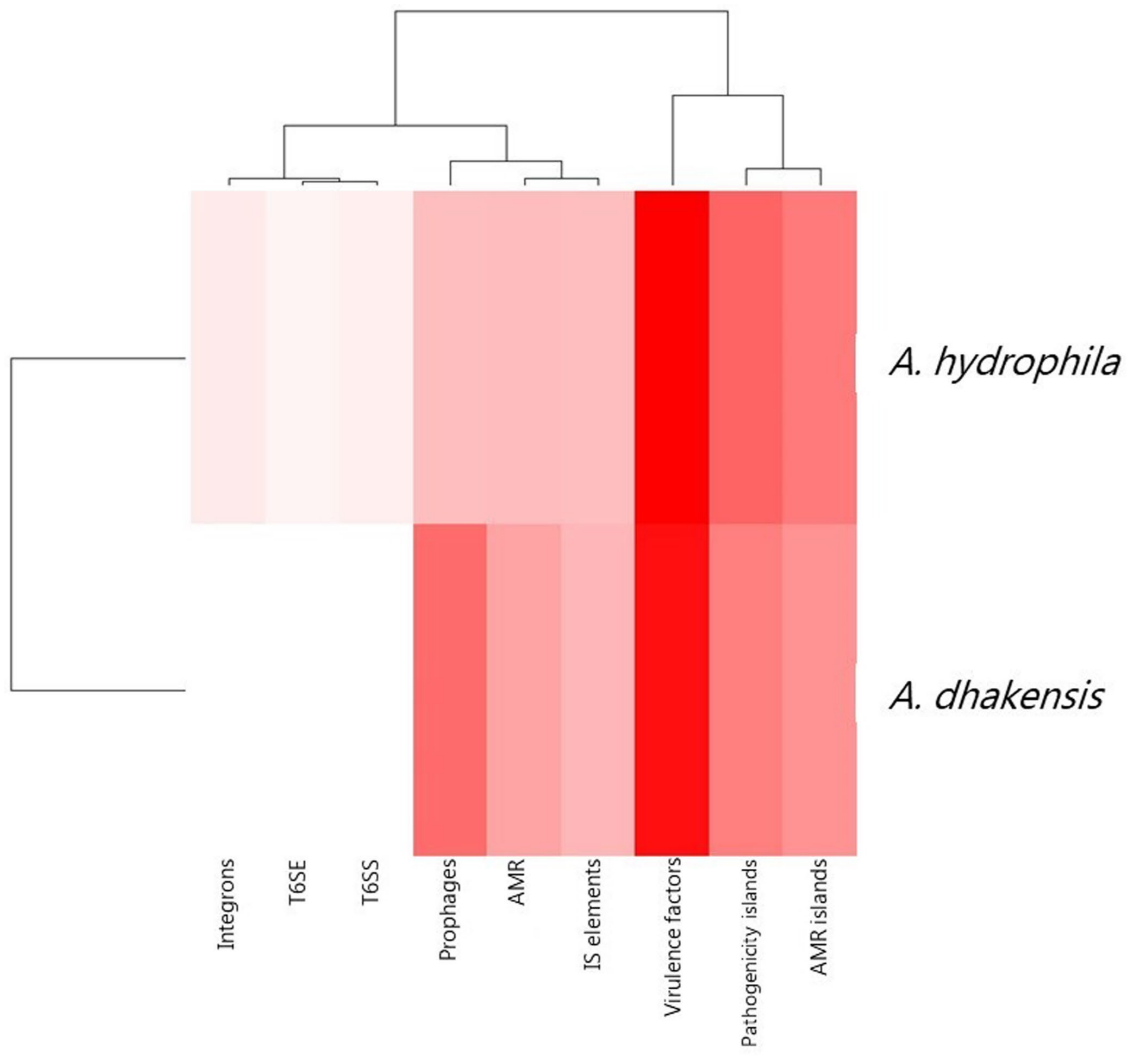
Unique attributes of *A. dhakensis* and *A. hydrophila* genomes. AMR (antimicrobial resistance), AMR islands (antimicrobial resistance islands), IS elements (insertions sequence elements), T6SS (type VI secretion systems), T6SE (type VI secretion effectors). A dark red color indicates higher prevalence of the genomic feature and a lighter color indicates lower prevalence of the feature.

The pan genome analysis reviled a core genome consisting of 2,799 genes shared by both *A. hydrophila* and *A. dhakensis*. *A. dhakensis* genomes had 1,276 unique genes whil*e A. hydrophila* genomes had 1,357 unique genes (Figure 7). Analyzing the unique gene regions in VRprofile reviled that the unique region of *A. dhakensis* consisted of 28 genes related to virulence factors, 13 genes related to antimicrobial resistance, two genes related to the type VI secretion system and type VI secretion effectors, 18 prophage related genes, two integron genes, 10 insertions sequence elements, 15 pathogenicity island genes and 12 antimicrobial resistance island genes. In *A. hydrophila*, the unique genes consisted of 52 genes related to virulence factors, 13 genes related to antimicrobial resistance, 12 prophage related genes, two genes related to the type VI secretion system and one type VI secretion effector, two integron genes, 12 insertion sequence element genes, 31 pathogenicity island genes and 26 antimicrobial resistance island genes (Figure 8).

## 4. Discussion

*A. dhakensis* ST656 was the dominating species causing motile *Aeromonas* septicemia in the Mekong Delta in Vietnam during the studied time period. All confirmed *A. hydrophila* isolates in our study were from 2019, 2020 and 2021. Another study conducted in the Mekong Delta with isolates collected earlier (2013–2015) showed that *A. dhakensis* was more frequently isolated than *A. hydrophila* already at that time (Bartie et al., 2023). Whether there has been a shift from *A. hydrophila* towards *A. dhakensis* or if *A. dhakensis* has been the main causative agent of motile *Aeromonas* septicemia all along is unclear as current confirmed isolates (next-generation sequencing MLST and/or WGS) of both species only date back to 2013. Our results corroborate previous findings of *A. dhakensis* being the dominating causative agent of motile *Aeromonas* septicemia in striped catfish farming in the Mekong Delta. Knowledge about which of the two *Aeromonas* species is causing motile *Aeromonas* septicemia may be important if there are species-specific differences in their susceptibility to antimicrobial treatment, their virulence and immunogenicity.

The approved vaccine by PHARMAQ AS, Norway, to prevent motile *Aeromonas* septicemia in Vietnam contains formalin-inactivated cultures of *A. hydrophila* serotypes A and B. It is administered through intraperitoneal injection of fish (PHARMAQ, 2018). Since this vaccine is based on *A. hydrophila* strains it is important to know if the vaccine is equally efficient and effective in preventing disease and transmission of motile *Aeromonas* septicemia caused by *A. dhakensis*. We compared structures of the cell wall, that are relevant for the pathogenesis, to look for differences between *A. hydrophila* compared to *A. dhakensis* (Jimenez et al., 2008). Analysing the lipopolysaccharide region, we saw that the genes *kdkA*, *waaC*, *waaA*, *wahF*, *waaE*, *waaF* and *coaD* were identical in *A. hydrophila* and *A. dhakensis*, while there were variations in the region containing the genes *hldD*, *wahA*, *waaL*, *wahB*, *wahC* and *wahE*. There were differences in the presence of outer membrane proteins. Only two out of six outer membrane proteins were shared by both species. The genes coding for the other outer membrane proteins were present in either *A. dhakensis* or *A. hydrophila*. There were differences between *A. hydrophila* and *A. dhakensis* when it came to outer membrane structures relevant to antigen recognition in the host. To establish whether the only current approved vaccine is equally efficient and effective in preventing motile *Aeromonas* septicemia caused by *A. dhakensis* as *A. hydrophila*, experimental infection studies comparing the vaccine’s potential in preventing disease caused by the two bacterial species are needed.

Until the early 2000’s, *A. dhakensis* was not a distinct species (Huys et al., 2002). With modern molecular-based detection methods it is now possible to distinguish *A. dhakensis* from *A. hydrophila*. Whole genome sequencing and multilocus phylogenetic analysis are to date the most accurate methods of identification (Chen et al., 2016; Du et al., 2021), but such methods are not routinely used for diagnostic purposes in Vietnam and most other countries. The PCR primers initially used to identify the *A. hydrophila* is based on an article published in 1990 (Pollard et al., 1990), before *A. dhakensis* was identified as a distinct species. If these PCR primers are used in diagnostic laboratories to identify *A. dhakensis*, this species will routinely be misidentified. The same applies for the MALDI-TOF commercial databases as it does not include. *A. dhakensis*. Instead, the MALDI-TOF will identify the species on genus level only, or find the closest matching bacterial species, which in our case was *A. hydrophila*. Thus, the use of such existing identification methods will overestimate the true burden of *A. hydrophila* and the burden of *A. dhakensis* will be underestimated. There is a need to develop a species-specific PCR method that can differentiate *A. dhakensis* from *A. hydrophila* which can be used in laboratories for research and diagnostic purposes.

In Vietnamese fish production, the most commonly used antimicrobials are phenicols (florfenicol), tetracyclines (oxytetracycline) and sulfonamides (Luu et al., 2021). In striped catfish farming the most commonly used antimicrobial is florfenicol (Pham et al., 2023) and the aquaculture sector is said to be a genetic hotspot for gene transfer, including antimicrobial resistance genes (Watts et al., 2017). It was therefore no surprise to see a high prevalence (47–70%) of antimicrobial resistance genes for these three antimicrobial groups commonly used in the production. However, we noted differences between the *A. dhakensis* and *A. hydrophila* isolates regarding the presence of antimicrobial resistance genes and MIC values to the tested antimicrobials. The florfenicol resistance gene, *floR*, was only found in *A. dhakensis* (14/25) and these isolates had an MIC ≥8 mg/L for florfenicol while the *A. hydrophila* isolates had MIC values ≤1 mg/L for florfenicol. None of the *A. hydrophila* genomes carried the *sul2* gene. All of the *A. dhakensis* genomes carried the *cphA4* or *cphA3* gene, while *A. hydrophila* genomes carried *cphA2* instead. All of the isolates with an MIC ≥8 mg/L for tetracycline also carried the tetracycline resistance gene *tet(A)*.

We found differences in the antimicrobial susceptibility between *A. dhakensis* and *A. hydrophila* with higher MIC values for florfenicol and tetracyclines seen in *A. dhakensis*. Our findings indicate that the disease-causing organism could impact the choice of which antimicrobial to use. However, in our study we only tested five confirmed *A. hydrophila* isolates in the MIC analysis. Any differences in the antimicrobial susceptibility between the two *Aeromonas* spp. isolated from striped catfish and practical implications for treatment needs further studies. Pharmacokinetic studies to support development of antimicrobial treatment guidelines for motile *Aeromonas* septicemia are also needed.

A study comparing *Aeromonas* spp. from fish and human clinical isolates found that *A. dhakensis* carried more virulence genes compared to other *Aeromonas* spp. (*A. caviae*, *A. veronii* and *A. hydrophila*) (Wu et al., 2019). In our genome analysis, we found differences between the virulence genes present in the *A. dhakensis* genomes compared to the *A. hydrophila* genomes. But unlike reported by Wu et al. (2019), when we compared the unique genes from both bacterial species, *A. hydrophila* had more unique virulence genes than *A. dhakensis*. The five *A. hydrophila* genomes carried virulence genes coding for Flp type IV pili. None of the *A. dhakensis* genomes carried any genes coding for this type of pili. The *ahpA* gene was present in all *A. dhakensis*, but none of the *A. hydrophila* genomes. This protease has previously been associated with pathogenic strains of *Aeromonas* spp. (Zhu et al., 2007). The serine protease gene *ser* was present in all *A. hydrophila*, but none of the *A. dhakensis* genomes. The gene coding for heatstable cytotonic enterotoxin, *ast*, was only present in *A. dhakensis* genomes. The enterotoxin Act is one of the most important virulence factors in *A. dhakensis* (Rasmussen-Ivey et al., 2016) and the gene coding for this enterotoxin was present in all genomes, including *A. hydrophila* genomes. The T2SS is known to secrete the enterotoxin Act in *Aeromonas* spp. and is, together with T6SS, common in aquatic isolates (Rasmussen-Ivey et al., 2016). However, a difference in the geographical spread of T6SS has been suggested where a complete set of core genes has been observed in *A. hydrophila* isolates from Asia whereas isolates from the United States only carry remnants of this secretion system (Rasmussen-Ivey et al., 2016). The T6SS plays an important role in the bacteria’s evasion of the host immune system. This secretions system can translocate proteins and toxins into the cytosol of other cells (Wang et al., 2023). There was no difference in the protein secretions systems used by the two bacterial species and the T2SS and T6SS were found in all genomes, suggesting that these secretion systems play and important part in the pathogenicity of both *A. hydrophila* and *A. dhakensis* in Vietnamese striped catfish production.

## 5. Conclusion

Our study confirms that the dominating species causing outbreaks of motile *Aeromonas* septicemia in striped catfish fingerlings in the Mekong Delta from 2017 to 2021 was *A. dhakensis* ST656. *A. hydrophila* ST251 was also isolated from diseased striped catfish fingerlings. The true burden of *A. dhakensis* as an aquatic pathogen has been masked due to diagnostic methods unable of differentiating *A. dhakensis* from *A. hydrophila*. As vaccines are important preventive measures to reduce disease and needs for using antimicrobials, studies comparing the current vaccine’s efficiency and effectiveness in preventing infection with both *A. dhakensis* and *A. hydrophila* are needed.

## Data availability statement

The datasets presented in this study can be found in online repositories. The names of the repository/repositories and accession number(s) can be found in the article/Supplementary material.

## Author contributions

VE: Writing – original draft, Writing – review & editing. LK: Writing – review & editing. YH: Writing – review & editing. TD: Writing – review & editing. TP: Writing – review & editing. AD: Writing – review & editing.

## Funding

VE was partly supported through a PhD scholarship from the Sino-Danish Center, a University Partnership between Denmark and China. Financial support to collect and store isolates in Vietnam was received from KMP-Singapore.

## Conflict of interest

The authors declare that the research was conducted in the absence of any commercial or financial relationships that could be construed as a potential conflict of interest.

## Publisher’s note

All claims expressed in this article are solely those of the authors and do not necessarily represent those of their affiliated organizations, or those of the publisher, the editors and the reviewers. Any product that may be evaluated in this article, or claim that may be made by its manufacturer, is not guaranteed or endorsed by the publisher.

## Footnotes

^1^ https://cge.food.dtu.dk/

^2^ https://github.com/tseemann/snippy

^3^ https://server.gview.ca/
