# Supplementary figures and images for: Comparative genomic analysis of Aeromonas dhakensis and Aeromonas hydrophila from diseased striped catfish fingerlings cultured in Vietnam
Source: Front Microbiol. 2023 Sep 22;14:1254781. doi: 10.3389/fmicb.2023.1254781 (PMC10556525; doi:10.3389/fmicb.2023.1254781)

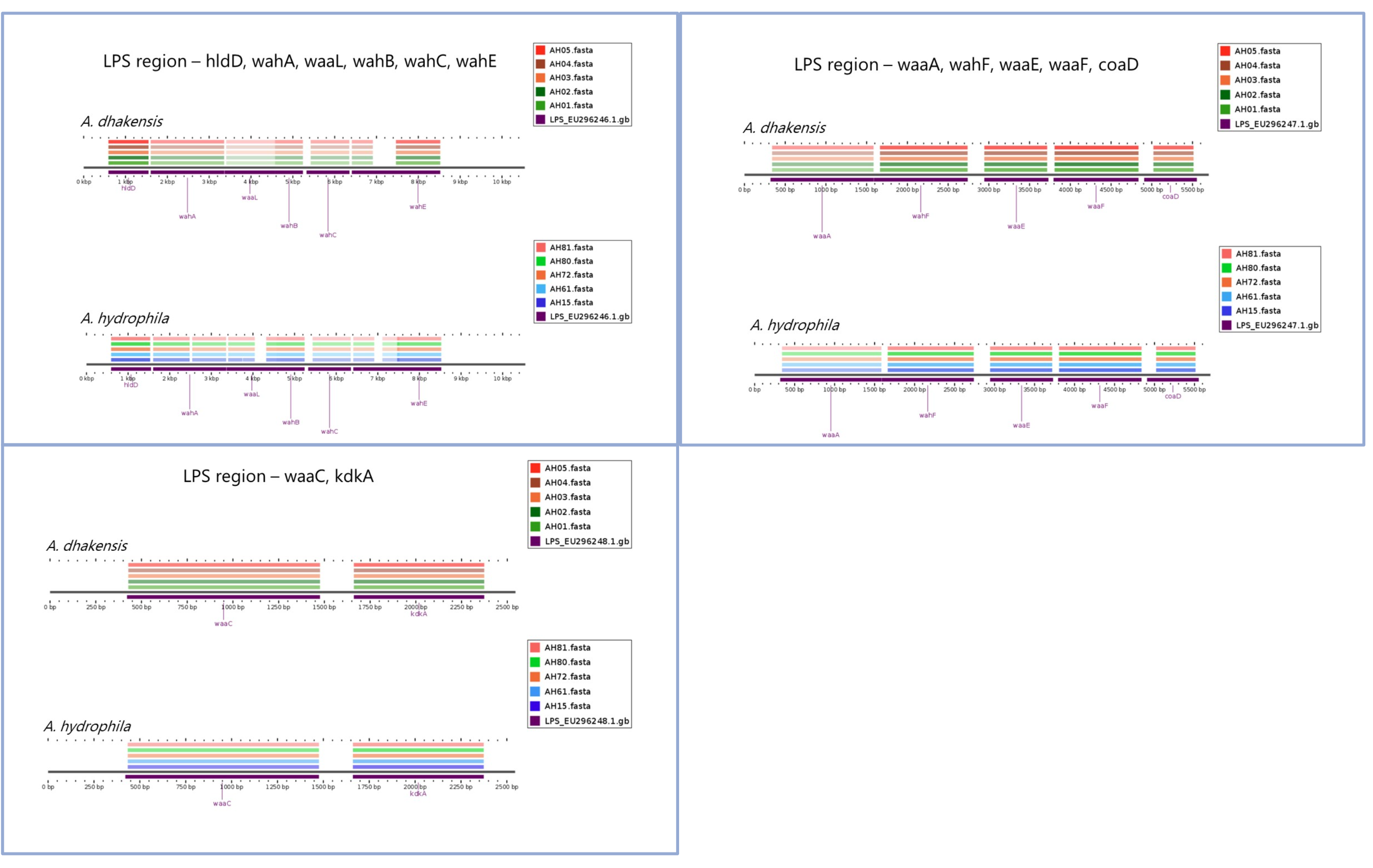

Supplement: Supplementary file 6 [file Image_1.PNG]
